# Transcript levels of Toll-Like receptors 5, 8 and 9 correlate with inflammatory activity in Ulcerative Colitis

**DOI:** 10.1186/1471-230X-11-138

**Published:** 2011-12-20

**Authors:** Fausto Sánchez-Muñoz, Gabriela Fonseca-Camarillo, Marco A Villeda-Ramírez, Elizabeth Miranda-Pérez, Edgar J Mendivil, Rafael Barreto-Zúñiga, Misael Uribe, Rafael Bojalil, Aarón Domínguez-López, Jesús K Yamamoto-Furusho

**Affiliations:** 1 Doctorado en Ciencias Biológicas y de la Salud, Universidad Autónoma Metropolitana, D.F., México; 2 Department of Gastroenterology, Instituto Nacional de Ciencias Médicas y Nutrición Salvador Zubiran, D. F., México; 3 Departament of Endoscopy, Instituto Nacional de Ciencias Médicas y Nutrición Salvador Zubiran, D. F., México; 4 Fundación Clínica Médica Sur, Puente de Piedra 150, D. F., México; 5 Department of Health Care. Universidad Autónoma Metropolitana, D.F., México; 6 Department of Immunology. Instituto Nacional de Cardiología "Ignacio Chávez", D. F., México

## Abstract

**Background:**

Dysregulation of innate immune response by Toll-Like Receptors (TLRs) is a key feature in Ulcerative Colitis (UC). Most studies have focused on *TLR2, TLR3*, and *TLR4 *participation in UC. However, few studies have explored other TLRs. Therefore, the aim of this study was to evaluate the mRNA profiles of *TLR1 to 9 *in colonic mucosa of UC patients, according to disease activity.

**Methods:**

Colonic biopsies were taken from colon during colonoscopy in 51 patients with Ulcerative Colitis and 36 healthy controls. mRNA levels of *TLR1 to 9, Tollip*, inflammatory cytokines *IL6 *and *TNF *were assessed by RT-qPCR with hydrolysis probes. Characterization of *TLR9 *protein expression was performed by Immunohistochemistry.

**Results:**

Toll-like receptors *TLR8, TLR9*, and *IL6 *mRNA levels were significantly higher in the colonic mucosa from UC patients (both quiescent and active) as compared to healthy individuals (p < 0.04). In the UC patients group the *TLR2, TLR4, TLR8 *and *TLR9 *mRNA levels were found to be significantly lower in patients with quiescent disease, as compared to those with active disease (p < 0.05), whereas *TLR5 *showed a trend (p = 0.06). *IL6 *and *TNF *mRNA levels were significantly higher in the presence of active disease and help to discriminate between quiescent and active disease (p < 0.05). Also, *IL6 *and *TNF *mRNA positively correlate with TLRs mRNA with the exception for *TLR3*, with stronger correlations for *TLR5, TLR8*, and *TLR9 *(p < 0.0001). *TLR9 *protein expression was mainly in the lamina propria infiltrate.

**Conclusions:**

This study demonstrates that *TLR2, TLR4, TLR8*, and *TLR9 *expression increases in active UC patients, and that the mRNA levels positively correlate with the severity of intestinal inflammation as well as with inflammatory cytokines.

## Background

Ulcerative colitis (UC) is a subtype of Inflammatory Bowel Disease (IBD) characterized by relapsing and chronic inflammation of colonic mucosa [1]. In Mexico, the frequency of new cases of UC has tripled during the last 20 years [2]. The etiology of UC is currently unknown, however inflammation is hypothesized to result from inappropriate activation of mucosal immunity by environmental factors such as gut microbiota [3]. Innate immunity mechanisms involved in recognition of microorganisms are thought implicated in many inflammatory conditions [4]. The Toll-like receptors (TLRs) have been clearly implicated in maintaining gut homeostasis and in the development of IBD [5] although the participation of the whole TLR family in the colonic mucosa from UC patients has not been fully explored.

TLRs are key regulators of the innate immune system in the gut through the induction of pro-inflammatory and immunomodulatory responses in many cell types including immune and epithelial cells [6]. *TLR1 *to *9 *have been reported detectable in human intestine at least in the mRNA levels both in healthy and disease conditions [5]. In particular *TLR2 *and *TLR4 *mRNA and protein have been reported to be up-regulated in IBD and in other intestinal inflammatory conditions [7-11]. In a recent study, published by Steenholdt, the *TLR8 *expression was found to be up-regulated in isolated cells from the colonic epithelial in patients with Crohn's disease and UC [12]. Also, very recently *TLR5 *expression was reported down-regulated in patients with UC [13].

To our knowledge, no previous studies have explored the mRNA expression of *TLR1 *to *9 *mRNA in the colonic biopsies from UC patients. Previous studies have focused mainly on the study of *TLR2, TLR3 *and, *TLR4*, however, other TLRs expression has not been extensively evaluated in UC patients. In gene expression studies, determination of mRNA levels by quantitative real time PCR (RT-qPCR) is a very robust method that has been used successfully in the IBD field [14,15]. Quantification of cytokines and chemokines mRNAs by this method has shown to be as an interesting tool to evaluate IBD disease activity [14,16,17]. RT-qPCR also has been traditionally used as gold standard to validate microarray results, due to its good variance coefficient in low level transcripts such as cytokines [18,19].

The aim of this study was to characterize the transcript patterns of *TLR1 *to *9 *in colonic biopsies from UC patients according to activity.

## Methods

### Population and tissue samples

A total of 87 individuals were studied and divided in 3 groups: 1) Active UC (n = 30); 2) Quiescent UC (n = 21) and Healthy control group (n = 36) table 1. All UC patients had a confirmed diagnosis of UC by histopathology and were recruited from the Inflammatory Bowel Disease Clinic at the National Institute of Medical Sciences between November 2007 and May 2009. Healthy controls consisted of those individuals who consent to colonoscopy for other reasons such as colorectal cancer screening, weight loss and anaemia. The inclusion criterion was histological normal findings on intestinal biopsy. Details of demographic and clinical characteristics of UC were obtained by a questionnaire, review of records and personal interview. Disease extension was defined by colonoscopy. The disease activity was determined by Mayo score and Riley criteria for endoscopic and histological activity respectively [20].

**Table 1:** Clinical and demographic characteristics of Ulcerative Colitis Patients

Patients Number Gender (M/F)	24/27
Age (years range)	41 (19-75)
Disease Duration (1-3/> 3 years)	10/40
Disease Activity (active/quiescent)	21/30
Disease Extension: distal colitis/Pancolitis	21/30
Endoscopic Activity (inactive/mild/moderate/severe)	20/8/12/11
Histological Activity (inactive/mild/moderate/severe)	13/16/12/10
Current therapy: **5**-aminosalicylate/Corticosteroids	23/18
Extra-intestinal Manifestations (without/arthritis/other)	31/18/8
Smoking habits (Current smoker/non-smoker/ex-smoker)	32/11/8
**Healthy Control Group**	
Number of Patients Sex (M/F)	14/22
Age (years range)	46 (18-64)

### Ethical considerations

This work was performed to the principles expressed in the Declaration of Helsinsky. This study was approved by the ethical committee in our hospital and a written informed consent was obtained from all patients and controls.

### Sample processing RNA extraction and cDNA synthesis

All 87 intestinal mucosal biopsies taken from colonoscopy were immediately placed in RNA later (Ambion, Austin, TX, USA) and stored at -70°C until processing. Then RNA isolation from all biopsies was performed with High Pure RNA Tissue Kit (ROCHE, Sciences, Maryland, USA) according to the manufacturer's instructions. The evaluation of RNA integrity, concentration, and purity was done by ribosomal visualizing RNA 18S and 28S integrity on agarose 1.5% gels, and by spectrophotometer on NanoDrop 2000 respectively (Thermo Fisher Scientific Lafayette, CO, USA). Duplicate cDNA synthesis was performed from 250 ng of total RNA using random hexamers and the Transcriptor first strand cDNA synthesis kit (ROCHE, Sciences, Maryland, USA).

### Real time RT-qPCR

RT-qPCR analysis was performed using Roche LightCycler 2.0 (ROCHE, Reuskreutz, Swisserland) with LNA hydrolysis probes from the Universal Probe Library Roche (UPL), and intron spanning designed primers (table 2) from Invitrogen (Carlsbad California, USA). One μl of cDNA was amplified with 200nM of primers, 100 nM of UPL probe, with the LighCycler TaqMan^® ^Master (ROCHE, Sciences, Maryland, USA) followed by 45 cycles of 95° 10 sec. 60° 30 sec., and 72° 1 sec. Reference genes *RPLP0, ACTB*, and *GAPDH *transcripts were used for relative quantification. For qPCR assays quality control, determination of linearity and reproducibility was evaluated (VC < 10%). The mRNA relative quantification of target genes was conducted using the LightCycler software 4.1, according to the 2-ΔΔCt method. The calibrator sample employed was the same patient sample performed in all runs.

**Table 2:** Primers Designs for qPCR

Gene	GENEBANK	PRIMERS (5'-3')	Amplicon Size (bp)	PROBE UPL
*TLR1*	NM_003263.3	CCTAGCAGTTATCACAAGCTCAAA	70	#79
		TCTTTTCCTTGGGCCATTC		
*TLR2*	NM_003264.3	CGTTCTCTCAGGTGACTGCTC	66	#14
		TCTCCTTTGGATCCTGCTTG		
*TLR3*	NM_003265.2	AGTTGTCATCGAATCAAATTAAAGAG	61	#80
		AATCTTCCAATTGCGTGAAAA		
*TLR4*	NM_138554.2	CTGCGTGAGACCAGAAAGC	75	#33
		TTCAGCTCCATGCATTGATAA		
*TLR5*	NM_003268.4	GACACAATCTCGGCTGACTG	105	#16
		TCAGGAACATGAACATCAATCTG		
*TLR6*	NM_006068.2	TGAAACAGTCTCTTTTGSGTAAATGC	72	#55
		CAGAATCCATTTGGGAAAGC		
*TLR7*	NM_016562.3	CCAGTGTCTAAAGAACCTGGAAA	63	#5
		TCAGGGACAGTGGTCAGTTG		
*TLR8*	NM_138636.2	AGCACTTCCCTCAGGAAGATT	62	#27
	NM_016610.2	AGCACCTTCAGATGAGGCATA		
*TLR9*	NM_017442.2	CCAGACCCTCTGGAGAAGC	133	#81
		GTAGGAAGGCAGGCAAGGT		
*TOLLIP*	NM_019009.2	TCCCCGCTGGAATAAGGT	95	#86
		CGTCCATGGAGAAGGCTCT		
*TNFA*	NM_000594.2	CAGCCTCTTCTCCTTCCTGA	123	#29
		GCCAGAGGGCTGATTAGAGA		
*IL6*	NM_000600	GCCCAGCTATGAACTCCTTCT	86	#45
		GAAGGCAGCAGGCAACAC		
*RPLP0	NM_001002.3	ACAGGGCGACCTGGAAGT	117	#32
	NM_053275.3	GGATCTGCTGCATCTGCTT		
*GADPH	NM_002046.3	AGCCACATCGCTCAGACAC	66	#60
		GCCCAATACGACCAAATCC		
*ACTB*	ENST00000331789.2	CAACCGCGAGAAGATGAC	121	560 nm
		GTCCATCACGATGCCAGT		

Note. *TLR8*, and *RPLP0 *assays were designed to detect both transcript isoforms, UPL (Universal Probe Library).

### Immunohistochemistry

Samples from six UC patients with and without inflammatory activity were included for TLR9 Immunohistochemistry. Peroxidase staining of paraffin-embedded tissue slides was performed using standard protocols. Briefly, after deparaffinizing and demasking of antigens, endogenous peroxidases were blocked with H2O2. Slides were blocked with 10% normal serum and were incubated with avidin and biotin. Following incubation with the primary antibody overnight at 4°C, slides were incubated with the secondary, biotin-conjugated antibody. Next, they were incubated with HRP-streptavidin, followed by incubation with the peroxidase substrate 3'-diaminobenzidine (DAB). In the negative controls, cells were stained omitting the primary antibody.

### Statistical Analysis

Statistical analysis was performed using SPSS Ver. 15 statistical package program. Statistical significance was considered when p value was < 0.05. Descriptive statistics were used as means and standard deviations and medians and interquartilar range. Kolmogorov-Smirnoff normality test determined RNA data distribution. Kruskal-Wallis and Mann Whitney U non parametric tests were used to test differences among groups, and Spearman correlation to asses the relationship between *TLR1 *to *9, TNF *and *IL6 *RNA levels and the endoscopic and histological parameters.

## Results

### *TLR1 *to *9 *mRNA profiles in UC compared with Controls

The *TLR1 *to *9*, TOLLIP, and *TNF *and *IL6 *mRNAs were detectable and quantifiable by RT-qPCR in intestinal biopsies from UC patients with quiescent and active disease as well as in healthy controls (table 3). *TLR4, TLR8*, and *TLR9 *mRNA levels were higher in the overall UC patients compared to healthy controls (p < 0.04) (table 3). We found a significantly increased expression of *TLR2, TLR4, TLR8*, and *TLR9 *mRNA expression in the colonic mucosa biopsies from patients with active UC compared with both quiescent UC and healthy controls (p ≤ 0.05) (table 3). Comparisons between the quiescent group and the control group, only showed a significant up-regulation for *TLR4 *mRNA levels (p = 0.04) (table 3), while the *TLR2, TLR8 *and *TLR9 *mRNA levels were similar in the healthy control and quiescent UC group (table 3). Interestingly, the *TLR5 *mRNA levels tend to be lower in the quiescent UC group as compared to active UC (p = 0.06) (table 3). The other TLRs (*TLR1, TLR3, TLR6 *and *TLR7*) and *TOLLIP *mRNA levels showed change in gene expression when comparing groups (p > 0.1) (table 3).

**Table 3:** Transcript levels of TLRs and pro-inflammatory cytokines in colonic mucosa from UC patients and Controls

GENE mRNA	Control N = 36	UC Patients N = 51	UC Quiescent N = 21	UC Active N = 31	Control vs UC	Control vs UC Quiescent	Control vs UC Active	UC Quiescent vs Active
	Transcript Levels				P value			
*TLR1*	0.75±0.50	1.12±1.63	1.33±2.51	0.98±0.53	0.26	0.64	0.13	0.11
*TLR2*	0.63±0.25	1.63±2.5	0.72±0.4	2.55±3.30	0.14	0.9	0.001	0.002
*TLR3*	0.54±0.25	0.75±0.5	0.87±0.60	0.63±0.39	0.6	0.18	0.7	0.27
*TLR4*	0.64±0.23	1.3±0.94	1.01±0.62	1.55±1.12	0.002	0.015	0.0001	0.04
*TLR5*	0.94±0.58	0.86±0.5	0.71±0.46	0.96±0.52	0.75	0.14	0.55	0.06*
*TLR6*	0.91±0.61	0.8±0.67	0.59±0.36	0.94±0.79	0.52	0.11	0.56	0.19
*TLR7*	0.71±0.45	0.62±0.79	0.77±1.21	0.52±0.27	0.86	0.14	0.13	0.82
*TLR8*	1.13±0.73	1.96±2.19	0.88±0.59	2.67±2.56	0.04	0.22	0.001	0.0005
*TLR9*	0.75±0.57	1.12±1.1	0.66±0.37	1.60±1.26	0.037	0.95	0.004	0.006
*TOLLIP*	0.79±0.21	0.84±0.28	0.85±0.32	0.83±0.26	0.41	0.69	0.82	0.86
*TNF*	0.96±1.28	0.84±0.79	0.4±0.37	1.24±0.86	0.96	0.01	0.023	< 0.001
*IL6*	1.8±3.5	8±19.5	1.6±3.2	11±23	0.039	0.8	< 0.001	< 0.001

Transcript levels are shown as means and standard deviations. P with significance are underlined and * for trend.

### Correlation of *TLR5, TLR8, TLR9*, and *IL6 *mRNA levels with endoscopic and histological activity

**Figure 1:**
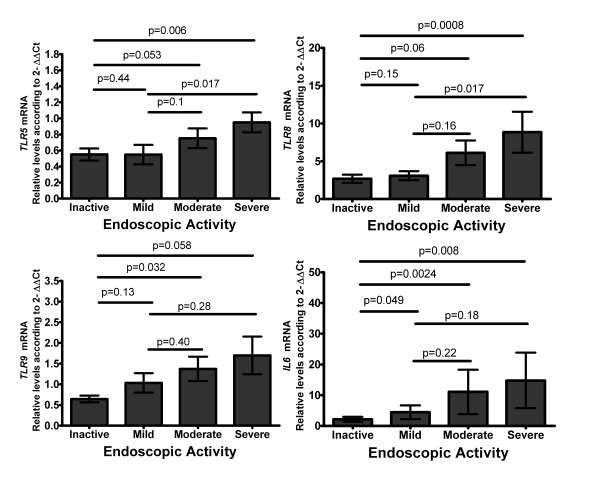
**Toll-like receptors *TLR5, TLR8*, and *TLR9 *mRNA levels in Ulcerative Colitis correlate with Endoscopic Activity**. RT-qPCR was performed to assess mRNA levels in colonic mucosa biopsies from UC patients according to Mayo subscore endoscopic activity, bars show means with standard error of the mean of *TLR*s and *IL6 *transcript levels with RPLP0 as housekeeping gene determined by 2^-ΔΔCt^, differences among groups were assessed by Mann-Whitney U test, and p values are presented in the figure.

**Figure 2:**
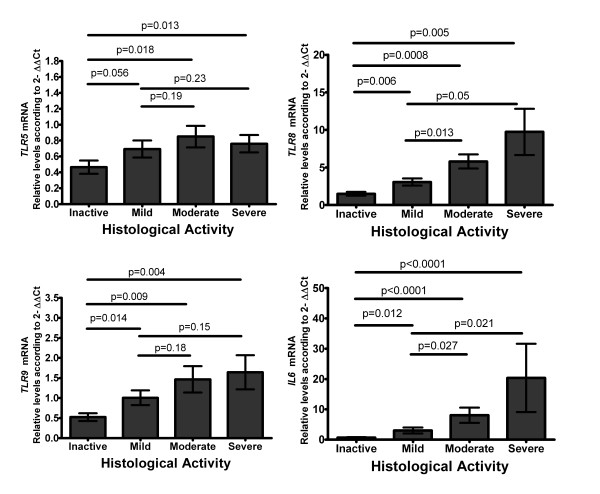
**Toll-like receptors *TLR5, TLR8*, and *TLR9 *mRNA levels in Ulcerative Colitis correlate with Histological Activity**. RT-qPCR was performed to assess mRNA levels in colonic mucosa biopsies from UC patients according to Riley Histological Activity, bars show means with standard error of the mean of *TLR*s and *IL6 *transcript levels with RPLP0 as housekeeping gene determined by 2^-ΔΔCt^, differences among groups were assessed by Mann-Whitney U test, and p values are presented in the figure.

To further evaluate the relation of less studied TLRs mRNA levels in IBD and gut inflammation in UC patients. We evaluated the correlation between *TLR5, TLR8, TLR9 *and *IL6 *mRNA expression levels and endoscopic (Figure 1) as well as histological activity (Figure 2) both activity scales were evaluated in a blinded fashion. Comparisons among groups are shown in the figures 1 and 2, *TLR5, TLR5 TLR5 *and *IL6 *mRNA levels were higher in the inflammatory active groups (p values are shown in the figures). Positive correlations were found for the mRNA levels of *TLR5 *(r = 0.387; p = 0.005), *TLR8 *(r = 0.465; p = 0.001), *TLR9 *(r = 0.288; p = 0.04), and *IL6 *(r = 0.439; p = 0.001) with endoscopic activity as shown in Figure 1. On the other hand, the mRNA levels of *TLR5, TLR8, TLR9*, and *IL6 *correlation with histological activity, such as *TLR5 *(r = 0.341; p = 0.015), *TLR8 *(r = 0.577; p < 0.001), *TLR9 *(r = 0.428; p = 0.002), and *IL6 *(r = 0.633; p < 0.001) as shown in Figure 2. The TLRs mRNA positive correlations with endoscopic and histological activity tend to be significantly higher analysing the subset of patients who did not received any steroid treatment at the moment of the study (overall r > 0.6; p < 0.01).

### TLRs mRNA levels correlate with *IL6*, and *TNF *mRNA levels in the colonic mucosa from UC patients

In this study, we also found that in particular *IL6 *mRNA levels strongly correlate with disease activity. The *IL6 *mRNA levels showed an important capacity to differentiate between quiescent and active disease (Receiver Operator Curve, AUC = 0.876; p < 0.0001) compared to *TNF *(Receiver Operator Curve, AUC = 0.700; p < 0.0001). Therefore, in we explore the correlation of *IL6 *and *TNF *mRNA levels as inflammatory markers with the TLRs mRNA levels. We found that *IL6 *and *TNF *mRNA levels showed a positive correlation with TLRs (*TLR1, TLR2, TLR4, TLR5, TLR6, TLR8 *and *TLR9*) (table 4). The most relevant association was found between *IL6 *and *TLR8 *mRNA levels showing also an r2 = 0.4626 p < 0.01 by linear regression analysis.

**Table 4:** Toll-like receptors and Tollip mRNA correlations with *IL6 *and *TNF *mRNAs in Rectum mucosa from UC patients

Gene Transcript	*IL6*		*TNF*	
	Rho	P	Rho	P
*TLR1*	0.589	< 0.001	0.572	< 0.001
*TLR2*	0.626	< 0.001	0.704	< 0.001
*TLR3*	-0.28	0.089	-0.069	0.679
*TLR4*	0.367	0.023	0.546	< 0.001
*TLR5*	0.565	< 0.001	0.615	< 0.001
*TLR6*	0.387	0.007	0.561	0.001
*TLR7*	0.34	0.05	0.242	0.168
*TLR8*	0.681	< 0.001	0.623	< 0.001
*TLR9*	0.583	< 0.001	0.643	< 0.001
*TOLLIP*	-0.264	0.11	-0.065	0.698

### Mucosa-Infiltrating Immune Cells Are a Major Source of Intestinal TLR9 Expression

**Figure 3:**
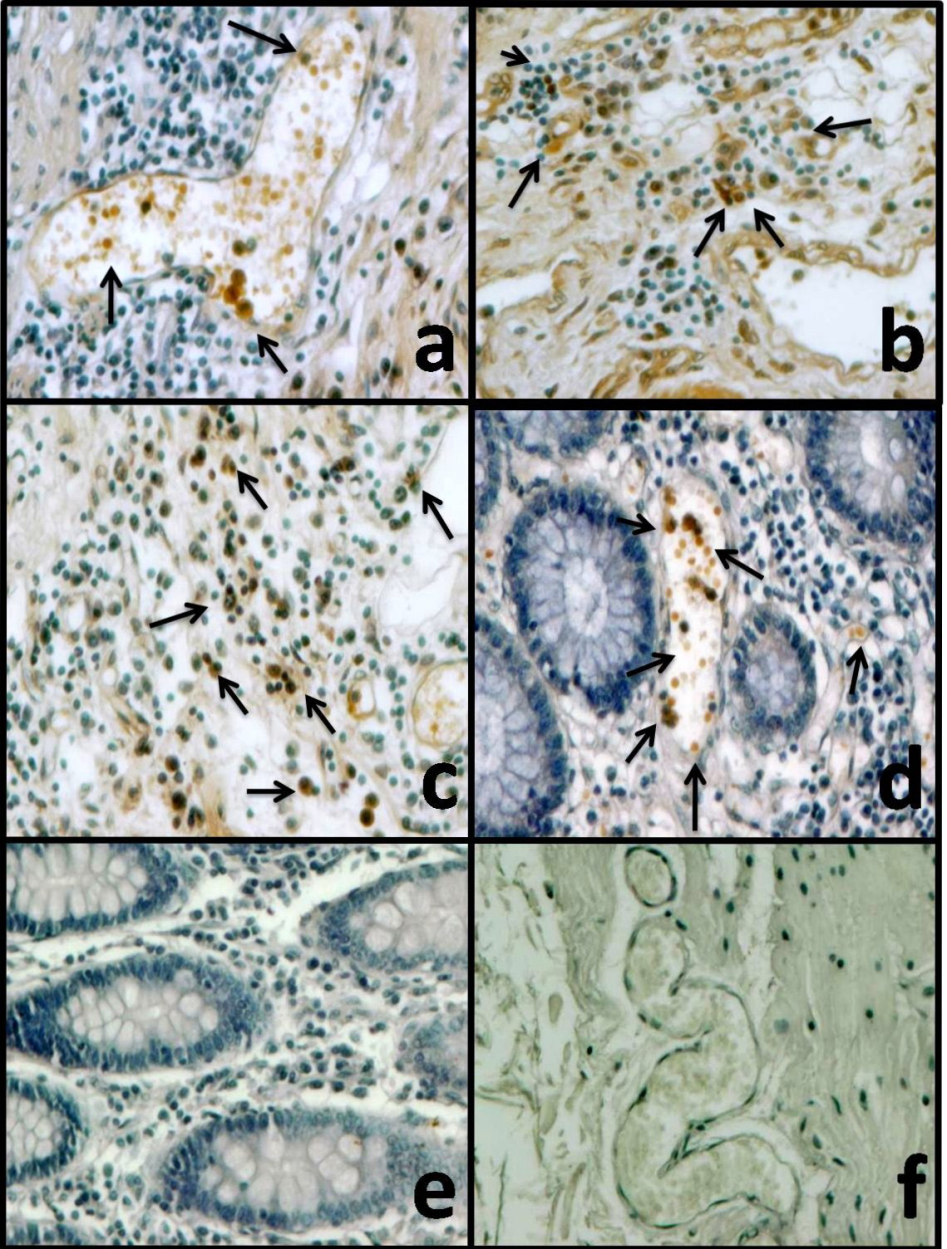
**TLR9 increased detection in positive infiltrating immune cells in active UC**. Representative immunoperoxidase analysis of TLR9 expression of inflamed colonic tissue from six patients (panel A-D) uninflamed colonic tissue (panel E-F) and taken from a representative patient with UC. In the negative controls (neg. ctrl), the secondary antibody was omitted. Original magnification was 20 × (panels A, C, D).

In order to further characterize the cells responsible for TLR9 expression, we determine in situ TLR9 protein expression from intestinal biopsies of UC patients, tissues were immunostained and compared with non-inflamed tissue (Figure 3e-f). The percentage of TLR9 immunoreactive cells was higher in UC patients compared to controls. TLR9+ cells were localized mainly in mucosa (Figure 3) lamina propria and perivascular inflammatory infiltrates (Figure 3d and 3e) but not in goblet cells, crypt lumen or crypt branching, neither submucosa.

## Discussion

In the present study, we found that gene expression of *TLR2, TLR4, TLR8 *and *TLR9 *was substantially up-regulated active UC patients. In particular, less studied *TLR5, TLR8 *and *TLR9 *gene expression positively correlated with the presence of UC and the severity of endoscopic and histological inflammation. The *IL6 *and *TNF *gene expression showed the same trend as TLRs. The other TLRs (*TLR1, TLR3, TLR6, TLR7)*, and *TOLLIP *mRNA levels were not found to be significantly altered in the presence of active UC. Our results corroborate previously reported results for *TLR2 *and *TLR4 *gene expression in IBD [7,8,11,21] and in general for intestinal inflammation such as Celiac Disease and IBS [9,22].

We found also that *TLR5 *mRNA tends to be up-regulated in active UC compared to UC quiescent disease (p = 0.06), but we also found that *TLR5 *also tends to be down-regulated in UC quiescent disease compared to healthy mucosa colonic controls (p = 0.14). In a previous study, Cario and Podolsky reported no changes of *TLR5 *expression in the colonic mucosa from IBD patients [7]. Stanislawowski and colleagues found a negative correlation between *TLR5 *and both macroscopically and microscopically inflammation [7,13]. We suspect that this different finding can be possibly due to a regulation of the *TLR5 *transcript levels by steroids therapy, because we do not found significant Spearman correlations of *TLR5 *mRNA and disease activity indexes (p > 0.1) compared to patients treated only with 5-ASA (p < 0.002). In addition, our results also show that TLR5 up-regulation is related to inflammation as seen in colectomized UC patients who developed pouchitis [23]. Also, Brint et al. recently found that *TLR5 *mRNA was up-regulated in colonic biopsies from active Irritable Bowel Syndrome patients [22].

The *TLR8 *gene expression in colonic mucosa determined by us confirmed the results published by Steenholdt et al. who showed that the *TLR8 *expression was highly up-regulated in colonic epithelial cells from active UC patients [12]. The functional consequence for *TLR8 *up-regulation in the gut is unknown, although except for *TLR2 *all other TLRs in epithelial cells induce pro-inflammatory signals [5]. *TLR8 *induction of IL8 by ligands in human isolated epithelial cells stimulated has been reported [12]. We suspect that *TLR8 *up-regulation in colonic epithelial cells may induce an exacerbated inflammatory response against not well studied gut microbiota virus or bacterial RNA resulting from microbiota dysbiosis.

Although for the case *TLR9*, expression was initially reported down-regulated in inflamed colonic mucosa from IBD patients [24]. On the other hand, we found that *TLR9 *gene expression was up-regulated in the presence of active UC and compared to healthy mucosa controls [25]. In the present study, we also found that *TLR9 *mRNA levels positively correlate with disease activity scores in a blinded fashion. Interestingly in a study conducted in the dog IBD form of disease colonic *TLR2, TLR4*, and *TLR9 *mRNA levels are up-regulated [26]. Also, for the *TLR9 *case we decide to corroborate protein expression by immunohistochemistry because of the differing results in determining the protein expression in colonic samples. We found that the most evident source of TLR9 up-regulation levels during inflammation were the mucosal infiltrating cells. In agreement with our results, Pedersen et al. reported very little *TLR9 *protein expression in isolated colonic epithelial cells assed by western blot [24]. To our knowledge, almost all other studies have analysed *TLR9 *expression in cell lines [27-29]. Our findings are relevant because previous a previous study reported by Hall et al. showed that Dendritic Cells signalling via *TLR9 *in response to commensal bacterial DNA inhibits Treg differentiation in the Gut [30]. Also, *in vitro TLR9 *expression has been reported induced by pathogenic bacterial DNA, in agreement to possible deleterious role of *TLR9 *signalling in the gut [27]. Finally, *TLR9 *frequent promoter polymorphisms associated with IBD might up-regulate *TLR9 *gene expression [31,32]. We believe that gut inflammation with leukocyte infiltration and dysbiosis both play a role in the up-regulation of *TLR9 *expression in the colonic mucosa of UC patients.

In order to evaluate the relation between inflammation and TLR gene expression, we also determine the correlations between *TLR1 *to *9 *mRNA expression and mRNA levels of pro-inflammatory cytokines such as *TNF *and *IL6 *(Table 4). It is well known that *TNF *and *IL6 *mRNA profiles positively correlate with inflammatory activity and especially *IL6 *can be used as activity marker in UC [16,17]. We also found that *TLR8 *mRNA levels are useful and efficient means to discriminate between active and quiescent UC.

Finally, the main limitations of our study are the lack of assessment of putative regulatory TLRs promoter polymorphisms, and the evaluation of colonic isolated epithelial and lamina propria isolated colonic cells will corroborate functional consequence of Toll-like receptors cross-talk.

## Conclusions

The *TLR5, TLR8 *and *TLR9 *gene expression were up-regulated in patients with active UC and had a positive correlation with endoscopic and histological activity. The functional consequence of *TLRs *over-expression in the mucosa from UC patients needs to be further clarified.

## Competing interests

The authors declare that they have no competing interests.

## Authors' contributions

All authors read and approved the final manuscript; FSM participate during the sample processing, performed RT-qPCR analysis for TLR mRNA levels and prepared the manuscript. GFC participate during the sample collecting, processing, RT-qPCR analysis for cytokine transcript level quantification and TLR9 Immunohistochemistry. MAVR participate during the sample recollecting, processing, and non-colitis control characterization. EMP participated during the sample recollecting and data analysis. EJM participate during the sample recollecting and data analysis. RBZ assessed clinical and endoscopic analysis and diagnostics and sampling procedures. MU did critical reviewing and guiding and data analysis. RB participated in critical reviewing and data discussion and analysis. ADL critical reviewing and bibliographic analysis, JKYF designed and provided the research idea, directed, reassessed both clinical and histological diagnostic, and coordinate the manuscript editing.

## Pre-publication history

The pre-publication history for this paper can be accessed here:

http://www.biomedcentral.com/1471-230X/11/138/prepub
